# Crystal structure of 5,15-dihexyl-5,15-di­hydro­benzo[2,1-*b*:4,3-*c*′]dicarbazole hexane 0.375-solvate

**DOI:** 10.1107/S2056989018014512

**Published:** 2018-10-19

**Authors:** Dingchao Zhang, Longqiang Shi, Zhi Liu, Li Li, Zhenhao Hu, Deliang Cui, Jing Yang

**Affiliations:** a State Key Laboratory of Crystal Materials, Shandong University, Jinan 250100, Shandong Province, People’s Republic of China; b School of Chemistry and Environmental Engineering, Shandong University of Science and Technology, Qingdao, People’s Republic of China

**Keywords:** crystal structure, π-conjugated, carbazole derivative, helicene, clathrate

## Abstract

The title compound, C_38_H_40_N_2_·0.375C_6_H_14_, crystallizes in the monoclinic space group *P*2_1_/*c* and has a host–guest structure with the helicene molecules forming a porous structure and mol­ecules of hexane inserted into the holes. The dihedral angles between the two carbazole sections of the right- and left-handed helicenes are 27.44 (3) and 25.63 (3)°, respectively. There are no classical π–π inter­actions or hydrogen-bonding inter­actions present between adjacent mol­ecules in the crystal structure. The hexane solvent mol­ecule shows positional disorder.

## Abstract 2

In the title compound, two helicenes form a porous structure with mol­ecules of hexane inserted into the holes.

## Chemical context   

π-conjugated organic mol­ecules have received a great deal of attention over the past few decades owing to their applications in organic field-effect transistors (Qi *et al.*, 2008 ▸; Upadhyay *et al.*, 2016 ▸) and organic light-emitting diodes (Hong *et al.*, 2016 ▸; Konidena *et al.*, 2015 ▸). 5,15-Dihexyl-5,15-di­hydro­benzo[2,1-*b*:4,3-*c*′]dicarbazole 0.375-hexane, **1**·0.375-hexane, with a carbazole unit as the primary building block was designed based on the following factors. Firstly, carbazole is a cheap chemical material with a rigid and planar structure, and high thermal and electrochemical stabilities (Konidena *et al.*, 2017 ▸). Secondly, introducing sufficient hexyl substituents to the helical core can enhance the solubility in common solvents drastically (Luo *et al.*, 2018 ▸) and suppress close-packing in the solid state (Chen *et al.*, 2017 ▸). Thirdly, a helical mol­ecular geometry results in a non-planar, twisted structure, which decreases mol­ecular aggregations and effectively hinders excited-state fluorescence quenching (Hua *et al.*, 2015 ▸; Shi *et al.*, 2012 ▸). Highly fused conjugated acenes can provide a high charge-carrier transport property as the conjugation length is increased (Pho *et al.*, 2012 ▸). Of these compounds, helicene derivatives have been extensively applied in mol­ecular recognition (Liu *et al.*, 2018 ▸) and in photoresponsive cholesteric liquid crystals (Kim *et al.*, 2017 ▸). As a result of their contribution to the development of chemical separations (Steed *et al.*, 1994 ▸), topochemical reactions (Toda, 1995 ▸), biomimicry (Ghadiri *et al.*, 1994 ▸) and so on, the design and synthesis of host–guest complexes has attracted intense inter­est. The recrystallization method provides a way of acquiring such complexes (Tanaka *et al.*, 2000 ▸; Tanaka *et al.*, 1995 ▸). By slow evaporation from a mixed solution of hexane and di­chloro­methane, we fortuitously obtained single crystals of the title compound **1**·0.375 hexane. Despite attempting to grow single crystals *via* several methods, we did not obtain any crystal structures of solvent-free host mol­ecules, indicating that inter­actions between host and guest mol­ecules are an important factor in crystal growth. 
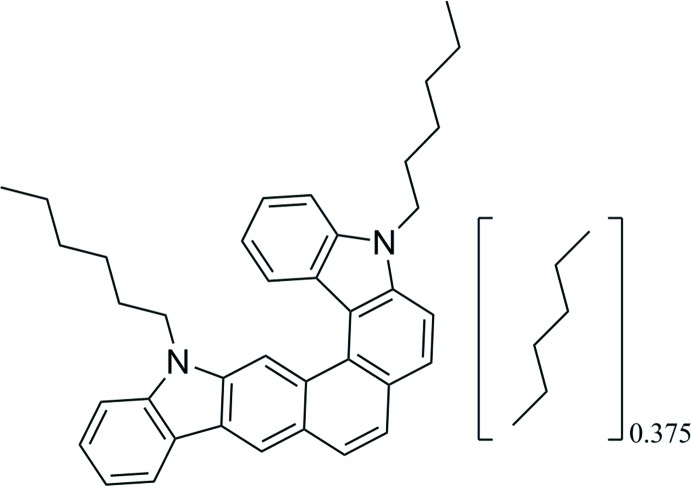


## Structural commentary   

**Figure 1:**
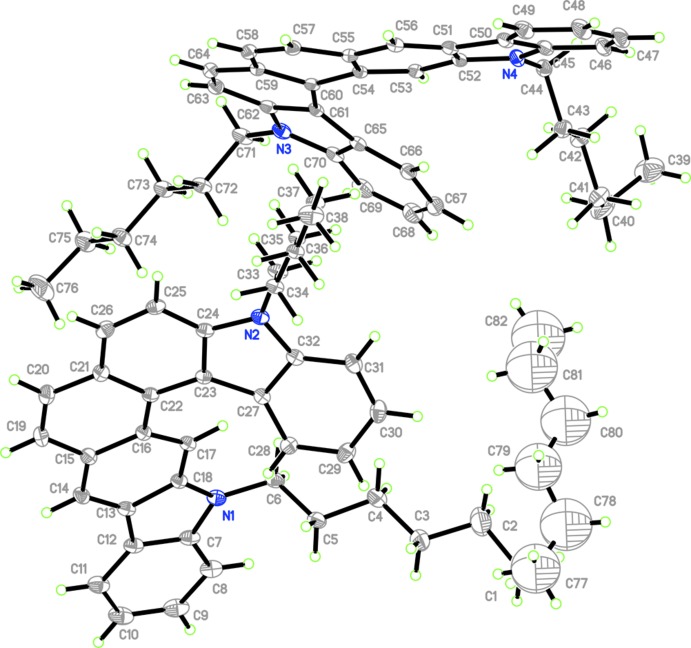
The mol­ecular structure of the title compound with the atom labelling. Displacement ellipsoids are drawn at the 30% probability level.

The title compound (Fig. 1 ▸) crystallizes in space group *P2_1_/c* with two chiral helicene mol­ecules and a partially occupied hexane mol­ecule in the unit cell. The host mol­ecule is a carbazole-based di­aza­[7]helicene whose geometrical parameters are similar to those of 5,15-dihexyl-5,15-di­hydro­benzo[2,1-*b*:4,3-c′]dicarbazole·(cyclo­hexa­ne)_0.5_ with a 2:1 stoichiometry of host and guest mol­ecules (Shi *et al.*, 2012 ▸). However, the proportion of host and guest mol­ecules in the title compound is 2:0.75 rather than 2:1, indicating that less hexane solvent is wrapped in the holes. In the right-handed helicene (containing N1, N2), the average C—C bond length [1.428 (3) Å] in the inner helical rim of the Fjord region [C16—C17 = 1.398 (3), C16—C22 =1.456 (3), C22—C23 = 1.429 (3), C23—C27 = 1.455 (3) and C27—C28 = 1.404 (3) Å] is increased by 0.033 Å relative to the average bond length [1.395 (3) Å] in aromatic compounds. The average of their counterparts [1.365 (3) Å] in the five peripheral rings [C13—C14 = 1.374 (3), C19—C20 = 1.345 (3), C25—C26 = 1.362 (3) and C30—C31 = 1.379 (3) Å] is decreased by 0.030 Å. In contrast, the average C—C bond length [1.431 (2) Å] in the inner helical rim of the Fjord region of the left-handed helicene (containing N3, N4) [C53—C54 = 1.407 (2), C54—C60 = 1.457 (2), C60—C61 = 1.430 (2), C61—C65 = 1.458 (3) and C65—C66 = 1.403 (3) Å] is increased by 0.036 Å while the average of their counterparts [1.364 (3) Å] in the five peripheral rings [C51—C56 = 1.375 (3), C57—C58 = 1.348 (3), C63—C64 = 1.358 (3) and C68—C69 = 1.374 (4) Å] is decreased by 0.031 Å. In the central ring, the C—C bond lengths in the right- and left-handed helicenes range from 1.345 (3) to 1.456 (3) Å and from 1.348 (3) to 1.457 (2) Å, respectively. The bond angles in the right- and left-handed helicenes are in the ranges 118.07 (17)–121.64 (19)° and 117.92 (16)–121.92 (17)°, respectively, indicating they are six-membered aromatic rings with a little distortion at C20 and C22, and C58 and C60. The dihedral angle between the two carbazole sections of the right- and left-handed helicenes are 27.44 (3) and 25.63 (3)°, respectively.

## Supra­molecular features   

**Figure 2:**
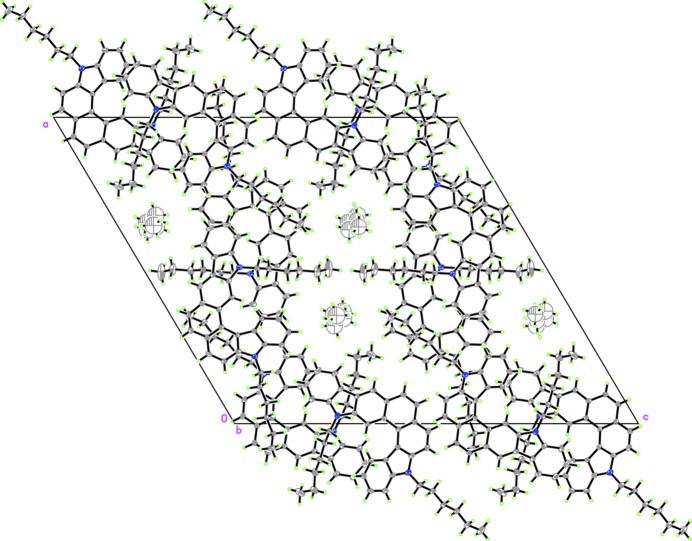
The crystal packing of the title compound viewed along the *b* axis.

The title mol­ecules are staggered and stacked in a face-to-face manner extending along the *b*-axis direction (see Fig. 2 ▸). The helicenes are packed forming a one-dimensional porous structure with hexane mol­ecules located in the holes. No classical π–π inter­actions or hydrogen bonding occur between adjacent mol­ecules because of the non-planar screw structure and the steric effects of long substituted hexyl chains.

## Database survey   

A search of the Cambridge Crystallagraphic Database (WebCSD, Version 1.1.2; last update May 2018) for 5,15-dihexyl-5,15-di­hydro­benzo[2,1-*b*:4,3-*c*′]dicarbazole, revealed 12 similar structures. The structure of carbazole-based 5,15-dihexyl-5,15-di­aza­[7]helicene and 7-hexyl-7-mono­aza­[6]helicene were elucidated and two regioisomeric phenalenocarbazoles were investigated by our research group (Hua *et al.*, 2015 ▸; Luo *et al.*, 2018 ▸; Shi *et al.*, 2012 ▸). Upadhyay *et al.* (2016 ▸) reported two different sites of aza­[*n*]helicene (*n*= 7 or 9) *via* photocyclization of bis-stillbene derivatives of carbazole leading to angular or linear structures. The crystal structures of aza-hepta­cenes based on an extended indolo[3,2-*b*]carbazole skeleton have been reported (Levick *et al.*, 2014 ▸). In the structure of carbazolo[4,3-*c*]carbazole, the packed mol­ecules are arranged in parallel planes (Más-Montoya *et al.*, 2013 ▸). In addition, several enantio-enriched aza­helicenes obtained *via* a Fischer indole reaction have been investigated (Kötzner *et al.*, 2014 ▸).

## Synthesis and crystallization   

**Figure 3:**
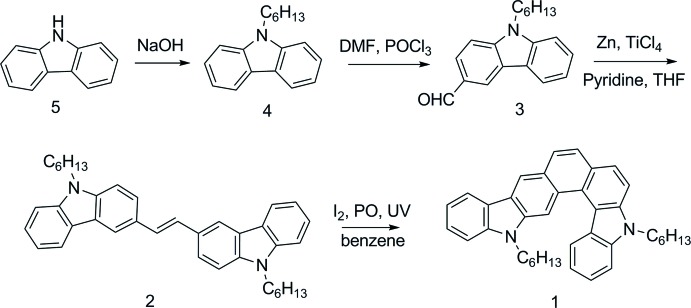
Reaction scheme.

All reactants and solvents were used as purchased without further purification while THF was refluxed with Na in the presence of benzo­phenone and DMF was dehydrated by using mol­ecular sieves. 9-Hexyl-9*H*-carbazole (**4**), 9-*H*exyl-9-carbazole-3-carbaldehyde (**3**), (*E*)-1,2-bis­(9-hexyl-9*H*-carbazol-3-yl)ethene (**2**) and 5,15-dihexyl-5,15-di­hydro­benzo[2,1-*b*:4,3-*c*′]dicarbazole (**1**) were synthesized according to the methods reported by our research group (Shi *et al.*, 2012 ▸) (see Fig. 3 ▸). Yellow block-shaped crystals were obtained from a mixed solution of di­chloro­methane/hexane (*v*:*v* = 1:1).

## Refinement details   

Crystal data, data collection and structure refinement details are summarized in Table 1 ▸. All H atoms were placed in geometrically calculated positions and refined using a riding model: C—H = 0.93–0.97Å (for CH_2_ groups) or 0.96 Å (for CH_3_ groups) with *U*_iso_(H) = 1.2*U*_eq_(C) or 1.5*U*_eq_(C-meth­yl). The hexane solvent mol­ecule shows positional disorder. The carbon atoms could not be determined reliably from the difference-Fourier map. They were refined at their found positions with isotropic displacement parameters, while C—C distances and C—C—C angles were restrained to target values of 1.500 (3)–1.521 (3) Å and 111.3 (2)–114.6 (2)°, respectively. The hexane solvent molecule has a refined occupancy of 0.751 (5).

## Supplementary Material

Crystal structure: contains datablock(s) I. DOI: 10.1107/S2056989018014512/ex2015sup1.cif

Structure factors: contains datablock(s) I. DOI: 10.1107/S2056989018014512/ex2015Isup2.hkl

CCDC reference: 1873208

Additional supporting information:  crystallographic information; 3D view; checkCIF report

## Figures and Tables

**Table 1:** Experimental details

Crystal data	
Chemical formula	C_38_H_40_N_2_·0.375C_6_H_14_
*M*_r_	557.05
Crystal system, space group	Monoclinic, *P*2_1_/*c*
Temperature (K)	130
*a*, *b*, *c* (Å)	28.446 (3), 8.2572 (8), 32.262 (3)
β (°)	120.602 (1)
*V* (Å^3^)	6522.4 (11)
*Z*	8
Radiation type	Mo *K*α
μ (mm^−1^)	0.07
Crystal size (mm)	0.48 × 0.47 × 0.08

Data collection	
Diffractometer	Bruker *APEX3* CCD area-detector
Absorption correction	Multi-scan (*SADABS*; Bruker, 2017 ▸)
*T*_min_, *T*_max_	0.676, 0.746
No. of measured, independent and observed [*I* > 2σ(*I*)] reflections	74304, 15039, 11584
*R*_int_	0.036
(sin θ/λ)_max_ (Å^−1^)	0.652

Refinement	
*R*[*F* ^2^ > 2σ(*F* ^2^)], *wR*(*F* ^2^), *S*	0.065, 0.191, 1.05
No. of reflections	15039
No. of parameters	751
No. of restraints	9
H-atom treatment	H-atom parameters constrained
Δρ_max_, Δρ_min_ (e Å^−3^)	0.66, −0.32

Computer programs: *APEX3* and *SAINT* (Bruker, 2017 ▸) and *SHELXTL* (Sheldrick, 2008 ▸).

## supplementary crystallographic information

### Crystal data

**Table:**

C_38_H_40_N_2_·0.375C_6_H_14_	*F*(000) = 2406
*M**_r_* = 557.04	*D*_x_ = 1.135 Mg m^−^^3^
Monoclinic, *P*2_1_/*c*	Mo *K*α radiation, λ = 0.71069 Å
Hall symbol: -P2xac	Cell parameters from 9166 reflections
*a* = 28.446 (3) Å	θ = 2.6–27.1°
*b* = 8.2572 (8) Å	µ = 0.07 mm^−^^1^
*c* = 32.262 (3) Å	*T* = 130 K
β = 120.602 (1)°	Block, yellow
*V* = 6522.4 (11) Å^3^	0.48 × 0.47 × 0.08 mm
*Z* = 8	

### Data collection

**Table:**

Bruker APEX3 CCD area-detector diffractometer	15039 independent reflections
Radiation source: fine-focus sealed tube	11584 reflections with *I* > 2σ(*I*)
Graphite monochromator	*R*_int_ = 0.036
φ and ω scans	θ_max_ = 27.6°, θ_min_ = 1.3°
Absorption correction: multi-scan (SADABS; Bruker, 2017)	*h* = −36→37
*T*_min_ = 0.676, *T*_max_ = 0.746	*k* = −10→10
74304 measured reflections	*l* = −41→41

### Refinement

**Table:**

Refinement on *F*^2^	Secondary atom site location: difference Fourier map
Least-squares matrix: full	Hydrogen site location: inferred from neighbouring sites
*R*[*F*^2^ > 2σ(*F*^2^)] = 0.065	H-atom parameters constrained
*wR*(*F*^2^) = 0.191	*w* = 1/[σ^2^(*F*_o_^2^) + (0.0922*P*)^2^ + 4.1418*P*] where *P* = (*F*_o_^2^ + 2*F*_c_^2^)/3
*S* = 1.05	(Δ/σ)_max_ = 0.001
15039 reflections	Δρ_max_ = 0.66 e Å^−^^3^
751 parameters	Δρ_min_ = −0.32 e Å^−^^3^
9 restraints	Extinction correction: SHELXTL (Bruker, 2017), Fc^*^=kFc[1+0.001xFc^2^λ^3^/sin(2θ)]^-1/4^
Primary atom site location: structure-invariant direct methods	Extinction coefficient: 0.0038 (4)

### Special details

**Table:**

Geometry. All esds (except the esd in the dihedral angle between two l.s. planes) are estimated using the full covariance matrix. The cell esds are taken into account individually in the estimation of esds in distances, angles and torsion angles; correlations between esds in cell parameters are only used when they are defined by crystal symmetry. An approximate (isotropic) treatment of cell esds is used for estimating esds involving l.s. planes.
Refinement. Refinement of F^2^ against ALL reflections. The weighted R-factor wR and goodness of fit S are based on F^2^, conventional R-factors R are based on F, with F set to zero for negative F^2^. The threshold expression of F^2^ > 2sigma(F^2^) is used only for calculating R-factors(gt) etc. and is not relevant to the choice of reflections for refinement. R-factors based on F^2^ are statistically about twice as large as those based on F, and R- factors based on ALL data will be even larger.

### Fractional atomic coordinates and isotropic or equivalent isotropic displacement parameters (Å^2^)

**Table:**

	*x*	*y*	*z*	*U*_iso_*/*U*_eq_	Occ. (<1)
C1	0.5055 (3)	−0.1727 (7)	0.4596 (2)	0.102 (3)	
H1A	0.4803	−0.2569	0.4408	0.254*	
H1B	0.5000	−0.1432	0.4856	0.254*	
H1C	0.5423	−0.2109	0.4724	0.254*	
C2	0.4959 (2)	−0.0236 (5)	0.42739 (14)	0.0973 (13)	
H2A	0.4600	0.0208	0.4166	0.117*	
H2B	0.5228	0.0591	0.4458	0.117*	
C3	0.50037 (13)	−0.0704 (3)	0.38431 (10)	0.0571 (7)	
H3A	0.4711	−0.1455	0.3647	0.068*	
H3B	0.5347	−0.1270	0.3956	0.068*	
C4	0.49766 (11)	0.0698 (3)	0.35284 (9)	0.0464 (5)	
H4A	0.5258	0.1478	0.3725	0.056*	
H4B	0.4626	0.1232	0.3398	0.056*	
C5	0.50520 (9)	0.0164 (3)	0.31151 (8)	0.0373 (5)	
H5A	0.5387	−0.0458	0.3243	0.045*	
H5B	0.4751	−0.0535	0.2902	0.045*	
C6	0.50778 (8)	0.1586 (2)	0.28295 (7)	0.0339 (4)	
H6A	0.4756	0.2255	0.2725	0.041*	
H6B	0.5395	0.2239	0.3039	0.041*	
C7	0.55750 (8)	0.0626 (2)	0.24130 (8)	0.0334 (4)	
C8	0.61069 (8)	0.0471 (3)	0.28015 (9)	0.0402 (5)	
H8	0.6194	0.0746	0.3112	0.048*	
C9	0.64987 (9)	−0.0107 (3)	0.27071 (10)	0.0478 (6)	
H9	0.6855	−0.0232	0.2961	0.057*	
C10	0.63758 (9)	−0.0504 (3)	0.22469 (10)	0.0486 (6)	
H10	0.6651	−0.0883	0.2197	0.058*	
C11	0.58477 (9)	−0.0347 (3)	0.18565 (9)	0.0419 (5)	
H11	0.5767	−0.0610	0.1547	0.050*	
C12	0.54424 (8)	0.0217 (2)	0.19433 (8)	0.0335 (4)	
C13	0.48572 (8)	0.0446 (2)	0.16383 (7)	0.0298 (4)	
C14	0.44926 (8)	0.0319 (2)	0.11526 (8)	0.0328 (4)	
H14	0.4614	0.0008	0.0946	0.039*	
C15	0.39362 (8)	0.0659 (2)	0.09664 (7)	0.0311 (4)	
C16	0.37415 (7)	0.1044 (2)	0.12878 (7)	0.0267 (4)	
C17	0.41204 (7)	0.1273 (2)	0.17767 (7)	0.0266 (4)	
H17	0.4006	0.1624	0.1985	0.032*	
C18	0.46662 (7)	0.0973 (2)	0.19462 (7)	0.0270 (4)	
C19	0.35664 (9)	0.0750 (3)	0.04594 (7)	0.0380 (5)	
H19	0.3686	0.0466	0.0250	0.046*	
C20	0.30472 (9)	0.1239 (3)	0.02798 (7)	0.0381 (5)	
H20	0.2827	0.1402	−0.0050	0.046*	
C21	0.28244 (8)	0.1517 (2)	0.05844 (7)	0.0316 (4)	
C22	0.31587 (7)	0.1296 (2)	0.10893 (7)	0.0266 (4)	
C23	0.28983 (7)	0.1388 (2)	0.13680 (6)	0.0254 (4)	
C24	0.23611 (7)	0.2004 (2)	0.11500 (7)	0.0277 (4)	
C25	0.20447 (8)	0.2310 (2)	0.06546 (7)	0.0327 (4)	
H25	0.1691	0.2708	0.0518	0.039*	
C26	0.22730 (8)	0.2004 (3)	0.03802 (7)	0.0348 (4)	
H26	0.2060	0.2119	0.0048	0.042*	
C27	0.30340 (7)	0.0900 (2)	0.18498 (7)	0.0262 (4)	
C28	0.34401 (8)	−0.0065 (2)	0.22151 (7)	0.0313 (4)	
H28	0.3724	−0.0470	0.2182	0.038*	
C29	0.34181 (9)	−0.0412 (3)	0.26227 (8)	0.0402 (5)	
H29	0.3687	−0.1056	0.2862	0.048*	
C30	0.29943 (9)	0.0196 (3)	0.26799 (8)	0.0426 (5)	
H30	0.2996	−0.0011	0.2964	0.051*	
C31	0.25751 (8)	0.1095 (3)	0.23230 (8)	0.0356 (4)	
H31	0.2292	0.1485	0.2360	0.043*	
C32	0.25913 (7)	0.1399 (2)	0.19054 (7)	0.0283 (4)	
C33	0.16650 (7)	0.2685 (2)	0.13886 (8)	0.0316 (4)	
H33A	0.1561	0.3615	0.1176	0.038*	
H33B	0.1701	0.3047	0.1690	0.038*	
C34	0.12106 (7)	0.1430 (2)	0.11627 (7)	0.0280 (4)	
H34A	0.1318	0.0475	0.1366	0.034*	
H34B	0.1156	0.1111	0.0852	0.034*	
C35	0.06763 (7)	0.2086 (2)	0.10983 (7)	0.0272 (4)	
H35A	0.0736	0.2416	0.1410	0.033*	
H35B	0.0571	0.3039	0.0895	0.033*	
C36	0.02106 (7)	0.0868 (2)	0.08768 (7)	0.0286 (4)	
H36A	0.0311	−0.0070	0.1086	0.034*	
H36B	0.0159	0.0509	0.0570	0.034*	
C37	−0.03285 (8)	0.1544 (2)	0.07970 (8)	0.0330 (4)	
H37A	−0.0283	0.1853	0.1105	0.040*	
H37B	−0.0421	0.2513	0.0600	0.040*	
C38	−0.07966 (9)	0.0344 (3)	0.05533 (10)	0.0495 (6)	
H38A	−0.0840	0.0020	0.0250	0.074*	
H38B	−0.1127	0.0843	0.0501	0.074*	
H38C	−0.0718	−0.0590	0.0755	0.074*	
C39	0.22389 (14)	0.6535 (5)	0.43881 (12)	0.0836 (11)	
H39A	0.2012	0.7457	0.4230	0.125*	
H39B	0.2614	0.6869	0.4567	0.125*	
H39C	0.2137	0.6066	0.4604	0.125*	
C40	0.21638 (12)	0.5258 (4)	0.40046 (10)	0.0708 (9)	
H40A	0.2257	0.5748	0.3782	0.085*	
H40B	0.2414	0.4365	0.4164	0.085*	
C41	0.15839 (11)	0.4602 (3)	0.37221 (9)	0.0515 (6)	
H41A	0.1570	0.3731	0.3514	0.062*	
H41B	0.1484	0.4158	0.3945	0.062*	
C42	0.11780 (9)	0.5895 (3)	0.34200 (8)	0.0425 (5)	
H42A	0.1327	0.6510	0.3257	0.051*	
H42B	0.1134	0.6632	0.3632	0.051*	
C43	0.06222 (9)	0.5266 (2)	0.30477 (7)	0.0360 (4)	
H43A	0.0462	0.4677	0.3206	0.043*	
H43B	0.0659	0.4524	0.2833	0.043*	
C44	0.02502 (8)	0.6659 (2)	0.27591 (7)	0.0320 (4)	
H44A	0.0168	0.7292	0.2968	0.038*	
H44B	0.0445	0.7357	0.2655	0.038*	
C45	−0.07269 (8)	0.5677 (2)	0.23389 (7)	0.0318 (4)	
C46	−0.08072 (10)	0.5500 (3)	0.27296 (8)	0.0387 (5)	
H46	−0.0533	0.5754	0.3041	0.046*	
C47	−0.13110 (11)	0.4930 (3)	0.26333 (10)	0.0475 (6)	
H47	−0.1374	0.4790	0.2887	0.057*	
C48	−0.17247 (10)	0.4561 (3)	0.21676 (10)	0.0475 (6)	
H48	−0.2059	0.4187	0.2115	0.057*	
C49	−0.16433 (9)	0.4744 (3)	0.17820 (9)	0.0389 (5)	
H49	−0.1923	0.4512	0.1470	0.047*	
C50	−0.11368 (8)	0.5282 (2)	0.18673 (7)	0.0313 (4)	
C51	−0.09004 (7)	0.5497 (2)	0.15655 (7)	0.0271 (4)	
C52	−0.03523 (7)	0.6019 (2)	0.18752 (7)	0.0260 (4)	
C53	0.00029 (7)	0.6303 (2)	0.17110 (6)	0.0249 (4)	
H53	0.0356	0.6663	0.1921	0.030*	
C54	−0.01778 (7)	0.6039 (2)	0.12215 (6)	0.0239 (4)	
C55	−0.07409 (7)	0.5634 (2)	0.09000 (6)	0.0257 (4)	
C56	−0.10903 (7)	0.5346 (2)	0.10810 (7)	0.0284 (4)	
H56	−0.1452	0.5051	0.0872	0.034*	
C57	−0.09496 (8)	0.5611 (2)	0.03913 (7)	0.0297 (4)	
H57	−0.1307	0.5278	0.0183	0.036*	
C58	−0.06321 (8)	0.6069 (2)	0.02131 (7)	0.0309 (4)	
H58	−0.0785	0.6138	−0.0119	0.037*	
C59	−0.00647 (8)	0.6452 (2)	0.05207 (7)	0.0277 (4)	
C60	0.01802 (7)	0.6283 (2)	0.10268 (6)	0.0242 (4)	
C61	0.07614 (7)	0.6429 (2)	0.13074 (7)	0.0251 (4)	
C62	0.10413 (8)	0.7049 (2)	0.10852 (7)	0.0284 (4)	
C63	0.07852 (8)	0.7353 (2)	0.05893 (7)	0.0332 (4)	
H63	0.0977	0.7790	0.0453	0.040*	
C64	0.02464 (8)	0.6984 (2)	0.03160 (7)	0.0326 (4)	
H64	0.0076	0.7083	−0.0017	0.039*	
C65	0.11858 (7)	0.5984 (2)	0.17940 (7)	0.0266 (4)	
C66	0.12071 (8)	0.5037 (2)	0.21644 (7)	0.0316 (4)	
H66	0.0887	0.4631	0.2136	0.038*	
C67	0.17044 (9)	0.4710 (3)	0.25716 (8)	0.0413 (5)	
H67	0.1717	0.4072	0.2814	0.050*	
C68	0.21895 (9)	0.5324 (3)	0.26237 (8)	0.0462 (6)	
H68	0.2518	0.5132	0.2908	0.055*	
C69	0.21901 (8)	0.6207 (3)	0.22630 (8)	0.0399 (5)	
H69	0.2514	0.6598	0.2296	0.048*	
C70	0.16890 (8)	0.6498 (2)	0.18457 (7)	0.0309 (4)	
C71	0.20084 (8)	0.7756 (2)	0.13177 (9)	0.0365 (5)	
H71A	0.2322	0.8118	0.1617	0.044*	
H71B	0.1865	0.8685	0.1105	0.044*	
C72	0.21961 (8)	0.6503 (2)	0.10887 (8)	0.0331 (4)	
H72A	0.1888	0.6162	0.0783	0.040*	
H72B	0.2335	0.5560	0.1296	0.040*	
C73	0.26400 (8)	0.7182 (2)	0.10073 (8)	0.0356 (4)	
H73A	0.2498	0.8129	0.0802	0.043*	
H73B	0.2944	0.7532	0.1315	0.043*	
C74	0.28513 (9)	0.6000 (3)	0.07802 (9)	0.0397 (5)	
H74A	0.2983	0.5037	0.0980	0.048*	
H74B	0.2550	0.5678	0.0468	0.048*	
C75	0.33021 (10)	0.6664 (3)	0.07165 (10)	0.0479 (6)	
H75A	0.3613	0.6922	0.1031	0.058*	
H75B	0.3179	0.7663	0.0533	0.058*	
C76	0.34851 (14)	0.5503 (4)	0.04613 (14)	0.0758 (9)	
H76A	0.3627	0.4534	0.0649	0.114*	
H76B	0.3765	0.6006	0.0422	0.114*	
H76C	0.3179	0.5235	0.0151	0.114*	
C77	0.3579 (6)	−0.4179 (13)	0.4050 (4)	0.275 (7)*	0.751 (5)
H77A	0.3763	−0.5048	0.4273	0.413*	0.751 (5)
H77B	0.3719	−0.4076	0.3837	0.413*	0.751 (5)
H77C	0.3194	−0.4406	0.3867	0.413*	0.751 (5)
C78	0.3672 (6)	−0.2610 (14)	0.4327 (3)	0.309 (9)*	0.751 (5)
H78A	0.4058	−0.2344	0.4494	0.370*	0.751 (5)
H78B	0.3565	−0.2752	0.4566	0.370*	0.751 (5)
C79	0.3347 (5)	−0.1224 (12)	0.3992 (3)	0.251 (6)*	0.751 (5)
H79A	0.3450	−0.1115	0.3749	0.301*	0.751 (5)
H79B	0.2962	−0.1502	0.3828	0.301*	0.751 (5)
C80	0.3426 (6)	0.0395 (13)	0.4240 (3)	0.284 (8)*	0.751 (5)
H80A	0.3254	0.0353	0.4433	0.340*	0.751 (5)
H80B	0.3814	0.0568	0.4457	0.340*	0.751 (5)
C81	0.3199 (5)	0.1814 (13)	0.3904 (4)	0.302 (8)*	0.751 (5)
H81A	0.2829	0.2014	0.3831	0.362*	0.751 (5)
H81B	0.3184	0.1543	0.3605	0.362*	0.751 (5)
C82	0.3532 (6)	0.3352 (14)	0.4106 (6)	0.326 (9)*	0.751 (5)
H82A	0.3367	0.4211	0.3876	0.489*	0.751 (5)
H82B	0.3897	0.3172	0.4173	0.489*	0.751 (5)
H82C	0.3541	0.3645	0.4399	0.489*	0.751 (5)
N1	0.51079 (6)	0.1120 (2)	0.24120 (6)	0.0313 (4)	
N2	0.21950 (6)	0.21004 (19)	0.14828 (6)	0.0292 (3)	
N3	0.15924 (7)	0.71767 (19)	0.14186 (6)	0.0316 (4)	
N4	−0.02603 (7)	0.6163 (2)	0.23392 (6)	0.0303 (3)	

### Atomic displacement parameters (Å^2^)

**Table:**

	*U*^11^	*U*^22^	*U*^33^	*U*^12^	*U*^13^	*U*^23^
C1	0.172 (9)	0.098 (4)	0.096 (4)	0.017 (5)	0.106 (4)	0.010 (3)
C2	0.168 (4)	0.075 (2)	0.089 (3)	0.003 (3)	0.095 (3)	−0.001 (2)
C3	0.0786 (19)	0.0488 (15)	0.0526 (15)	0.0033 (13)	0.0399 (14)	−0.0017 (12)
C4	0.0539 (14)	0.0425 (13)	0.0459 (13)	0.0058 (11)	0.0277 (11)	−0.0055 (10)
C5	0.0383 (11)	0.0329 (11)	0.0390 (11)	0.0043 (9)	0.0184 (9)	−0.0040 (9)
C6	0.0306 (10)	0.0282 (10)	0.0358 (10)	0.0009 (8)	0.0118 (8)	−0.0067 (8)
C7	0.0270 (9)	0.0230 (9)	0.0499 (12)	−0.0011 (7)	0.0194 (9)	0.0022 (8)
C8	0.0292 (10)	0.0319 (11)	0.0522 (13)	−0.0025 (8)	0.0154 (10)	0.0012 (9)
C9	0.0262 (10)	0.0405 (12)	0.0688 (16)	0.0006 (9)	0.0183 (11)	0.0052 (11)
C10	0.0319 (11)	0.0416 (12)	0.0799 (18)	0.0031 (9)	0.0339 (12)	0.0040 (12)
C11	0.0371 (11)	0.0349 (11)	0.0641 (15)	0.0020 (9)	0.0335 (11)	0.0048 (10)
C12	0.0295 (10)	0.0241 (9)	0.0512 (12)	−0.0006 (7)	0.0238 (9)	0.0042 (8)
C13	0.0290 (9)	0.0223 (9)	0.0452 (11)	−0.0005 (7)	0.0239 (9)	0.0017 (8)
C14	0.0374 (10)	0.0291 (10)	0.0438 (11)	−0.0009 (8)	0.0292 (9)	−0.0006 (8)
C15	0.0352 (10)	0.0275 (9)	0.0369 (10)	−0.0029 (8)	0.0229 (9)	−0.0016 (8)
C16	0.0273 (9)	0.0235 (9)	0.0323 (9)	−0.0023 (7)	0.0173 (8)	−0.0005 (7)
C17	0.0262 (9)	0.0229 (9)	0.0339 (9)	−0.0019 (7)	0.0177 (8)	−0.0024 (7)
C18	0.0276 (9)	0.0212 (8)	0.0347 (10)	−0.0021 (7)	0.0176 (8)	−0.0004 (7)
C19	0.0439 (12)	0.0435 (12)	0.0341 (10)	−0.0027 (9)	0.0253 (10)	−0.0044 (9)
C20	0.0422 (11)	0.0432 (12)	0.0276 (10)	−0.0027 (9)	0.0168 (9)	−0.0019 (9)
C21	0.0319 (10)	0.0304 (10)	0.0308 (10)	−0.0028 (8)	0.0148 (8)	−0.0012 (8)
C22	0.0255 (9)	0.0224 (8)	0.0309 (9)	−0.0018 (7)	0.0136 (8)	−0.0016 (7)
C23	0.0237 (8)	0.0207 (8)	0.0300 (9)	−0.0022 (7)	0.0122 (7)	−0.0027 (7)
C24	0.0246 (9)	0.0214 (8)	0.0366 (10)	−0.0024 (7)	0.0152 (8)	−0.0018 (7)
C25	0.0240 (9)	0.0296 (10)	0.0376 (10)	0.0023 (7)	0.0107 (8)	0.0035 (8)
C26	0.0302 (10)	0.0351 (10)	0.0290 (10)	−0.0018 (8)	0.0077 (8)	0.0011 (8)
C27	0.0225 (8)	0.0257 (9)	0.0307 (9)	−0.0048 (7)	0.0137 (7)	−0.0046 (7)
C28	0.0253 (9)	0.0341 (10)	0.0340 (10)	−0.0016 (8)	0.0148 (8)	0.0011 (8)
C29	0.0326 (10)	0.0480 (13)	0.0368 (11)	−0.0010 (9)	0.0154 (9)	0.0086 (10)
C30	0.0391 (11)	0.0571 (14)	0.0361 (11)	−0.0082 (10)	0.0224 (10)	0.0007 (10)
C31	0.0304 (10)	0.0417 (11)	0.0407 (11)	−0.0060 (8)	0.0224 (9)	−0.0058 (9)
C32	0.0243 (9)	0.0255 (9)	0.0357 (10)	−0.0046 (7)	0.0156 (8)	−0.0049 (7)
C33	0.0250 (9)	0.0249 (9)	0.0456 (11)	0.0003 (7)	0.0184 (8)	−0.0039 (8)
C34	0.0263 (9)	0.0227 (9)	0.0380 (10)	−0.0003 (7)	0.0186 (8)	−0.0029 (7)
C35	0.0269 (9)	0.0224 (9)	0.0341 (9)	0.0012 (7)	0.0169 (8)	−0.0006 (7)
C36	0.0266 (9)	0.0245 (9)	0.0373 (10)	0.0011 (7)	0.0181 (8)	−0.0001 (7)
C37	0.0293 (10)	0.0296 (10)	0.0434 (11)	0.0032 (8)	0.0210 (9)	0.0013 (8)
C38	0.0301 (11)	0.0455 (13)	0.0710 (17)	−0.0028 (10)	0.0242 (11)	−0.0010 (12)
C39	0.069 (2)	0.078 (2)	0.0624 (19)	0.0107 (17)	0.0042 (16)	0.0028 (17)
C40	0.0524 (16)	0.092 (2)	0.0505 (16)	0.0141 (16)	0.0135 (13)	0.0246 (16)
C41	0.0564 (15)	0.0507 (14)	0.0424 (13)	0.0084 (12)	0.0216 (12)	0.0123 (11)
C42	0.0444 (12)	0.0363 (11)	0.0373 (11)	−0.0041 (9)	0.0138 (10)	0.0030 (9)
C43	0.0442 (12)	0.0273 (10)	0.0361 (11)	−0.0048 (8)	0.0202 (9)	−0.0005 (8)
C44	0.0399 (11)	0.0272 (9)	0.0297 (10)	−0.0048 (8)	0.0184 (9)	−0.0053 (8)
C45	0.0390 (11)	0.0240 (9)	0.0427 (11)	0.0026 (8)	0.0284 (9)	−0.0002 (8)
C46	0.0518 (13)	0.0335 (11)	0.0441 (12)	0.0013 (9)	0.0340 (11)	−0.0032 (9)
C47	0.0623 (15)	0.0426 (13)	0.0636 (15)	0.0017 (11)	0.0510 (14)	−0.0004 (11)
C48	0.0489 (13)	0.0448 (13)	0.0702 (16)	−0.0036 (11)	0.0458 (13)	−0.0045 (12)
C49	0.0361 (11)	0.0360 (11)	0.0540 (13)	−0.0018 (9)	0.0297 (10)	−0.0041 (9)
C50	0.0352 (10)	0.0244 (9)	0.0426 (11)	0.0019 (8)	0.0259 (9)	−0.0010 (8)
C51	0.0263 (9)	0.0220 (8)	0.0369 (10)	0.0018 (7)	0.0190 (8)	−0.0001 (7)
C52	0.0294 (9)	0.0205 (8)	0.0295 (9)	0.0025 (7)	0.0161 (8)	−0.0006 (7)
C53	0.0248 (8)	0.0212 (8)	0.0292 (9)	0.0004 (7)	0.0141 (7)	−0.0019 (7)
C54	0.0256 (9)	0.0181 (8)	0.0284 (9)	0.0035 (6)	0.0140 (7)	0.0008 (7)
C55	0.0257 (9)	0.0216 (8)	0.0290 (9)	0.0030 (7)	0.0133 (7)	0.0002 (7)
C56	0.0244 (9)	0.0241 (9)	0.0356 (10)	0.0008 (7)	0.0146 (8)	−0.0015 (7)
C57	0.0254 (9)	0.0296 (9)	0.0283 (9)	0.0047 (7)	0.0094 (8)	−0.0003 (7)
C58	0.0318 (10)	0.0319 (10)	0.0252 (9)	0.0083 (8)	0.0118 (8)	0.0026 (7)
C59	0.0323 (9)	0.0230 (9)	0.0300 (9)	0.0061 (7)	0.0175 (8)	0.0021 (7)
C60	0.0259 (9)	0.0188 (8)	0.0297 (9)	0.0029 (7)	0.0153 (7)	−0.0004 (7)
C61	0.0282 (9)	0.0198 (8)	0.0315 (9)	0.0009 (7)	0.0183 (8)	−0.0036 (7)
C62	0.0298 (9)	0.0191 (8)	0.0428 (11)	0.0010 (7)	0.0232 (9)	−0.0025 (7)
C63	0.0406 (11)	0.0268 (9)	0.0446 (11)	0.0043 (8)	0.0306 (10)	0.0041 (8)
C64	0.0414 (11)	0.0299 (10)	0.0329 (10)	0.0075 (8)	0.0236 (9)	0.0048 (8)
C65	0.0236 (8)	0.0247 (9)	0.0314 (9)	−0.0002 (7)	0.0139 (7)	−0.0071 (7)
C66	0.0293 (9)	0.0351 (10)	0.0309 (10)	0.0034 (8)	0.0157 (8)	−0.0028 (8)
C67	0.0355 (11)	0.0536 (14)	0.0312 (10)	0.0105 (10)	0.0144 (9)	0.0006 (9)
C68	0.0290 (10)	0.0628 (15)	0.0357 (11)	0.0095 (10)	0.0085 (9)	−0.0088 (11)
C69	0.0244 (9)	0.0454 (12)	0.0469 (12)	−0.0023 (9)	0.0160 (9)	−0.0153 (10)
C70	0.0291 (9)	0.0268 (9)	0.0400 (11)	−0.0010 (7)	0.0199 (9)	−0.0097 (8)
C71	0.0350 (10)	0.0253 (10)	0.0609 (13)	−0.0052 (8)	0.0331 (10)	−0.0078 (9)
C72	0.0296 (10)	0.0250 (9)	0.0514 (12)	−0.0024 (7)	0.0255 (9)	−0.0041 (8)
C73	0.0348 (10)	0.0274 (10)	0.0536 (12)	−0.0022 (8)	0.0291 (10)	−0.0023 (9)
C74	0.0374 (11)	0.0372 (11)	0.0528 (13)	−0.0008 (9)	0.0290 (10)	−0.0041 (10)
C75	0.0485 (13)	0.0457 (13)	0.0656 (16)	−0.0031 (11)	0.0407 (13)	−0.0007 (11)
C76	0.081 (2)	0.076 (2)	0.111 (3)	−0.0040 (17)	0.078 (2)	−0.0127 (19)
N1	0.0246 (8)	0.0287 (8)	0.0376 (9)	0.0000 (6)	0.0136 (7)	−0.0017 (7)
N2	0.0230 (7)	0.0281 (8)	0.0366 (9)	0.0004 (6)	0.0152 (7)	−0.0014 (7)
N3	0.0291 (8)	0.0261 (8)	0.0453 (9)	−0.0025 (6)	0.0231 (8)	−0.0051 (7)
N4	0.0359 (9)	0.0289 (8)	0.0323 (8)	−0.0025 (7)	0.0218 (7)	−0.0040 (7)

### Geometric parameters (Å, º)

**Table:**

C1—C2	1.543 (6)	C42—C43	1.509 (3)
C1—H1A	0.9600	C42—H42A	0.9700
C1—H1B	0.9600	C42—H42B	0.9700
C1—H1C	0.9600	C43—C44	1.519 (3)
C2—C3	1.508 (4)	C43—H43A	0.9700
C2—H2A	0.9700	C43—H43B	0.9700
C2—H2B	0.9700	C44—N4	1.452 (2)
C3—C4	1.515 (4)	C44—H44A	0.9700
C3—H3A	0.9700	C44—H44B	0.9700
C3—H3B	0.9700	C45—N4	1.386 (2)
C4—C5	1.519 (3)	C45—C46	1.399 (3)
C4—H4A	0.9700	C45—C50	1.405 (3)
C4—H4B	0.9700	C46—C47	1.385 (3)
C5—C6	1.517 (3)	C46—H46	0.9300
C5—H5A	0.9700	C47—C48	1.391 (4)
C5—H5B	0.9700	C47—H47	0.9300
C6—N1	1.445 (3)	C48—C49	1.385 (3)
C6—H6A	0.9700	C48—H48	0.9300
C6—H6B	0.9700	C49—C50	1.395 (3)
C7—N1	1.388 (2)	C49—H49	0.9300
C7—C8	1.395 (3)	C50—C51	1.448 (3)
C7—C12	1.404 (3)	C51—C56	1.375 (3)
C8—C9	1.381 (3)	C51—C52	1.424 (3)
C8—H8	0.9300	C52—C53	1.379 (3)
C9—C10	1.381 (4)	C52—N4	1.387 (2)
C9—H9	0.9300	C53—C54	1.407 (2)
C10—C11	1.391 (3)	C53—H53	0.9300
C10—H10	0.9300	C54—C55	1.436 (2)
C11—C12	1.398 (3)	C54—C60	1.457 (2)
C11—H11	0.9300	C55—C56	1.406 (3)
C12—C13	1.452 (3)	C55—C57	1.433 (3)
C13—C14	1.374 (3)	C56—H56	0.9300
C13—C18	1.422 (3)	C57—C58	1.348 (3)
C14—C15	1.406 (3)	C57—H57	0.9300
C14—H14	0.9300	C58—C59	1.435 (3)
C15—C19	1.427 (3)	C58—H58	0.9300
C15—C16	1.438 (3)	C59—C64	1.417 (3)
C16—C17	1.398 (3)	C59—C60	1.419 (3)
C16—C22	1.456 (3)	C60—C61	1.430 (2)
C17—C18	1.381 (3)	C61—C62	1.411 (3)
C17—H17	0.9300	C61—C65	1.458 (3)
C18—N1	1.389 (2)	C62—N3	1.382 (2)
C19—C20	1.345 (3)	C62—C63	1.402 (3)
C19—H19	0.9300	C63—C64	1.358 (3)
C20—C21	1.433 (3)	C63—H63	0.9300
C20—H20	0.9300	C64—H64	0.9300
C21—C26	1.416 (3)	C65—C66	1.403 (3)
C21—C22	1.419 (3)	C65—C70	1.419 (3)
C22—C23	1.429 (3)	C66—C67	1.381 (3)
C23—C24	1.412 (2)	C66—H66	0.9300
C23—C27	1.455 (3)	C67—C68	1.398 (3)
C24—N2	1.378 (2)	C67—H67	0.9300
C24—C25	1.401 (3)	C68—C69	1.374 (4)
C25—C26	1.362 (3)	C68—H68	0.9300
C25—H25	0.9300	C69—C70	1.396 (3)
C26—H26	0.9300	C69—H69	0.9300
C27—C28	1.404 (3)	C70—N3	1.380 (3)
C27—C32	1.421 (3)	C71—N3	1.458 (2)
C28—C29	1.378 (3)	C71—C72	1.518 (3)
C28—H28	0.9300	C71—H71A	0.9700
C29—C30	1.402 (3)	C71—H71B	0.9700
C29—H29	0.9300	C72—C73	1.522 (3)
C30—C31	1.379 (3)	C72—H72A	0.9700
C30—H30	0.9300	C72—H72B	0.9700
C31—C32	1.394 (3)	C73—C74	1.516 (3)
C31—H31	0.9300	C73—H73A	0.9700
C32—N2	1.378 (2)	C73—H73B	0.9700
C33—N2	1.460 (2)	C74—C75	1.502 (3)
C33—C34	1.522 (3)	C74—H74A	0.9700
C33—H33A	0.9700	C74—H74B	0.9700
C33—H33B	0.9700	C75—C76	1.518 (4)
C34—C35	1.525 (2)	C75—H75A	0.9700
C34—H34A	0.9700	C75—H75B	0.9700
C34—H34B	0.9700	C76—H76A	0.9600
C35—C36	1.521 (3)	C76—H76B	0.9600
C35—H35A	0.9700	C76—H76C	0.9600
C35—H35B	0.9700	C77—C78	1.517 (3)
C36—C37	1.526 (3)	C77—H77A	0.9600
C36—H36A	0.9700	C77—H77B	0.9600
C36—H36B	0.9700	C77—H77C	0.9600
C37—C38	1.519 (3)	C78—C79	1.521 (3)
C37—H37A	0.9700	C78—H78A	0.9700
C37—H37B	0.9700	C78—H78B	0.9700
C38—H38A	0.9600	C79—C80	1.515 (3)
C38—H38B	0.9600	C79—H79A	0.9700
C38—H38C	0.9600	C79—H79B	0.9700
C39—C40	1.555 (5)	C80—C81	1.500 (3)
C39—H39A	0.9600	C80—H80A	0.9700
C39—H39B	0.9600	C80—H80B	0.9700
C39—H39C	0.9600	C81—C82	1.518 (4)
C40—C41	1.521 (4)	C81—H81A	0.9700
C40—H40A	0.9700	C81—H81B	0.9700
C40—H40B	0.9700	C82—H82A	0.9600
C41—C42	1.509 (3)	C82—H82B	0.9600
C41—H41A	0.9700	C82—H82C	0.9600
C41—H41B	0.9700		
			
C2—C1—H1A	109.5	C42—C43—C44	110.35 (17)
C2—C1—H1B	109.5	C42—C43—H43A	109.6
H1A—C1—H1B	109.5	C44—C43—H43A	109.6
C2—C1—H1C	109.5	C42—C43—H43B	109.6
H1A—C1—H1C	109.5	C44—C43—H43B	109.6
H1B—C1—H1C	109.5	H43A—C43—H43B	108.1
C3—C2—C1	110.3 (3)	N4—C44—C43	114.31 (16)
C3—C2—H2A	109.6	N4—C44—H44A	108.7
C1—C2—H2A	109.6	C43—C44—H44A	108.7
C3—C2—H2B	109.6	N4—C44—H44B	108.7
C1—C2—H2B	109.6	C43—C44—H44B	108.7
H2A—C2—H2B	108.1	H44A—C44—H44B	107.6
C2—C3—C4	115.0 (2)	N4—C45—C46	128.7 (2)
C2—C3—H3A	108.5	N4—C45—C50	109.53 (17)
C4—C3—H3A	108.5	C46—C45—C50	121.69 (19)
C2—C3—H3B	108.5	C47—C46—C45	117.3 (2)
C4—C3—H3B	108.5	C47—C46—H46	121.4
H3A—C3—H3B	107.5	C45—C46—H46	121.4
C3—C4—C5	112.6 (2)	C46—C47—C48	121.8 (2)
C3—C4—H4A	109.1	C46—C47—H47	119.1
C5—C4—H4A	109.1	C48—C47—H47	119.1
C3—C4—H4B	109.1	C49—C48—C47	120.7 (2)
C5—C4—H4B	109.1	C49—C48—H48	119.7
H4A—C4—H4B	107.8	C47—C48—H48	119.7
C6—C5—C4	112.39 (18)	C48—C49—C50	119.1 (2)
C6—C5—H5A	109.1	C48—C49—H49	120.5
C4—C5—H5A	109.1	C50—C49—H49	120.5
C6—C5—H5B	109.1	C49—C50—C45	119.46 (19)
C4—C5—H5B	109.1	C49—C50—C51	133.9 (2)
H5A—C5—H5B	107.9	C45—C50—C51	106.62 (17)
N1—C6—C5	113.84 (16)	C56—C51—C52	118.75 (17)
N1—C6—H6A	108.8	C56—C51—C50	134.81 (18)
C5—C6—H6A	108.8	C52—C51—C50	106.42 (16)
N1—C6—H6B	108.8	C53—C52—N4	128.84 (17)
C5—C6—H6B	108.8	C53—C52—C51	122.46 (17)
H6A—C6—H6B	107.7	N4—C52—C51	108.70 (16)
N1—C7—C8	128.9 (2)	C52—C53—C54	118.97 (17)
N1—C7—C12	109.71 (17)	C52—C53—H53	120.5
C8—C7—C12	121.35 (19)	C54—C53—H53	120.5
C9—C8—C7	117.5 (2)	C53—C54—C55	118.87 (16)
C9—C8—H8	121.3	C53—C54—C60	121.76 (16)
C7—C8—H8	121.3	C55—C54—C60	119.21 (16)
C10—C9—C8	121.9 (2)	C56—C55—C57	120.34 (17)
C10—C9—H9	119.1	C56—C55—C54	120.26 (17)
C8—C9—H9	119.1	C57—C55—C54	119.32 (16)
C9—C10—C11	121.2 (2)	C51—C56—C55	120.34 (17)
C9—C10—H10	119.4	C51—C56—H56	119.8
C11—C10—H10	119.4	C55—C56—H56	119.8
C10—C11—C12	118.1 (2)	C58—C57—C55	120.51 (18)
C10—C11—H11	121.0	C58—C57—H57	119.7
C12—C11—H11	121.0	C55—C57—H57	119.7
C11—C12—C7	120.1 (2)	C57—C58—C59	121.92 (17)
C11—C12—C13	133.3 (2)	C57—C58—H58	119.0
C7—C12—C13	106.60 (17)	C59—C58—H58	119.0
C14—C13—C18	119.02 (17)	C64—C59—C60	120.64 (18)
C14—C13—C12	134.64 (18)	C64—C59—C58	119.71 (17)
C18—C13—C12	106.29 (17)	C60—C59—C58	119.65 (17)
C13—C14—C15	120.28 (18)	C59—C60—C61	116.92 (16)
C13—C14—H14	119.9	C59—C60—C54	117.92 (16)
C15—C14—H14	119.9	C61—C60—C54	125.11 (16)
C14—C15—C19	120.86 (18)	C62—C61—C60	118.58 (17)
C14—C15—C16	119.94 (18)	C62—C61—C65	105.44 (16)
C19—C15—C16	119.03 (18)	C60—C61—C65	135.84 (17)
C17—C16—C15	119.00 (17)	N3—C62—C63	126.80 (17)
C17—C16—C22	121.85 (17)	N3—C62—C61	110.01 (17)
C15—C16—C22	119.00 (17)	C63—C62—C61	123.01 (18)
C18—C17—C16	119.21 (17)	C64—C63—C62	117.36 (18)
C18—C17—H17	120.4	C64—C63—H63	121.3
C16—C17—H17	120.4	C62—C63—H63	121.3
C17—C18—N1	128.92 (17)	C63—C64—C59	122.25 (18)
C17—C18—C13	122.08 (18)	C63—C64—H64	118.9
N1—C18—C13	109.00 (16)	C59—C64—H64	118.9
C20—C19—C15	121.10 (19)	C66—C65—C70	117.57 (17)
C20—C19—H19	119.4	C66—C65—C61	135.23 (17)
C15—C19—H19	119.4	C70—C65—C61	106.51 (16)
C19—C20—C21	121.64 (19)	C67—C66—C65	119.88 (19)
C19—C20—H20	119.2	C67—C66—H66	120.1
C21—C20—H20	119.2	C65—C66—H66	120.1
C26—C21—C22	120.46 (18)	C66—C67—C68	120.9 (2)
C26—C21—C20	119.87 (18)	C66—C67—H67	119.5
C22—C21—C20	119.67 (18)	C68—C67—H67	119.5
C21—C22—C23	117.16 (16)	C69—C68—C67	121.2 (2)
C21—C22—C16	118.07 (17)	C69—C68—H68	119.4
C23—C22—C16	124.75 (16)	C67—C68—H68	119.4
C24—C23—C22	118.82 (17)	C68—C69—C70	117.9 (2)
C24—C23—C27	105.27 (16)	C68—C69—H69	121.1
C22—C23—C27	135.80 (17)	C70—C69—H69	121.1
N2—C24—C25	127.05 (17)	N3—C70—C69	128.31 (18)
N2—C24—C23	110.24 (16)	N3—C70—C65	109.15 (17)
C25—C24—C23	122.46 (18)	C69—C70—C65	122.38 (19)
C26—C25—C24	117.79 (18)	N3—C71—C72	114.30 (16)
C26—C25—H25	121.1	N3—C71—H71A	108.7
C24—C25—H25	121.1	C72—C71—H71A	108.7
C25—C26—C21	122.15 (18)	N3—C71—H71B	108.7
C25—C26—H26	118.9	C72—C71—H71B	108.7
C21—C26—H26	118.9	H71A—C71—H71B	107.6
C28—C27—C32	117.53 (17)	C71—C72—C73	111.17 (16)
C28—C27—C23	135.35 (17)	C71—C72—H72A	109.4
C32—C27—C23	106.51 (16)	C73—C72—H72A	109.4
C29—C28—C27	120.08 (19)	C71—C72—H72B	109.4
C29—C28—H28	120.0	C73—C72—H72B	109.4
C27—C28—H28	120.0	H72A—C72—H72B	108.0
C28—C29—C30	120.7 (2)	C74—C73—C72	114.45 (17)
C28—C29—H29	119.6	C74—C73—H73A	108.6
C30—C29—H29	119.6	C72—C73—H73A	108.6
C31—C30—C29	121.2 (2)	C74—C73—H73B	108.6
C31—C30—H30	119.4	C72—C73—H73B	108.6
C29—C30—H30	119.4	H73A—C73—H73B	107.6
C30—C31—C32	117.65 (19)	C75—C74—C73	114.02 (18)
C30—C31—H31	121.2	C75—C74—H74A	108.7
C32—C31—H31	121.2	C73—C74—H74A	108.7
N2—C32—C31	128.23 (17)	C75—C74—H74B	108.7
N2—C32—C27	109.17 (16)	C73—C74—H74B	108.7
C31—C32—C27	122.50 (18)	H74A—C74—H74B	107.6
N2—C33—C34	114.17 (15)	C74—C75—C76	113.3 (2)
N2—C33—H33A	108.7	C74—C75—H75A	108.9
C34—C33—H33A	108.7	C76—C75—H75A	108.9
N2—C33—H33B	108.7	C74—C75—H75B	108.9
C34—C33—H33B	108.7	C76—C75—H75B	108.9
H33A—C33—H33B	107.6	H75A—C75—H75B	107.7
C33—C34—C35	111.59 (15)	C75—C76—H76A	109.5
C33—C34—H34A	109.3	C75—C76—H76B	109.5
C35—C34—H34A	109.3	H76A—C76—H76B	109.5
C33—C34—H34B	109.3	C75—C76—H76C	109.5
C35—C34—H34B	109.3	H76A—C76—H76C	109.5
H34A—C34—H34B	108.0	H76B—C76—H76C	109.5
C36—C35—C34	113.50 (15)	C78—C77—H77A	109.5
C36—C35—H35A	108.9	C78—C77—H77B	109.5
C34—C35—H35A	108.9	H77A—C77—H77B	109.5
C36—C35—H35B	108.9	C78—C77—H77C	109.5
C34—C35—H35B	108.9	H77A—C77—H77C	109.5
H35A—C35—H35B	107.7	H77B—C77—H77C	109.5
C35—C36—C37	113.42 (15)	C77—C78—C79	111.3 (2)
C35—C36—H36A	108.9	C77—C78—H78A	109.4
C37—C36—H36A	108.9	C79—C78—H78A	109.4
C35—C36—H36B	108.9	C77—C78—H78B	109.4
C37—C36—H36B	108.9	C79—C78—H78B	109.4
H36A—C36—H36B	107.7	H78A—C78—H78B	108.0
C38—C37—C36	112.90 (17)	C80—C79—C78	114.6 (2)
C38—C37—H37A	109.0	C80—C79—H79A	108.6
C36—C37—H37A	109.0	C78—C79—H79A	108.6
C38—C37—H37B	109.0	C80—C79—H79B	108.6
C36—C37—H37B	109.0	C78—C79—H79B	108.6
H37A—C37—H37B	107.8	H79A—C79—H79B	107.6
C37—C38—H38A	109.5	C81—C80—C79	114.5 (2)
C37—C38—H38B	109.5	C81—C80—H80A	108.6
H38A—C38—H38B	109.5	C79—C80—H80A	108.6
C37—C38—H38C	109.5	C81—C80—H80B	108.6
H38A—C38—H38C	109.5	C79—C80—H80B	108.6
H38B—C38—H38C	109.5	H80A—C80—H80B	107.6
C40—C39—H39A	109.5	C80—C81—C82	113.4 (3)
C40—C39—H39B	109.5	C80—C81—H81A	108.9
H39A—C39—H39B	109.5	C82—C81—H81A	108.9
C40—C39—H39C	109.5	C80—C81—H81B	108.9
H39A—C39—H39C	109.5	C82—C81—H81B	108.9
H39B—C39—H39C	109.5	H81A—C81—H81B	107.7
C41—C40—C39	112.7 (3)	C81—C82—H82A	109.5
C41—C40—H40A	109.1	C81—C82—H82B	109.5
C39—C40—H40A	109.1	H82A—C82—H82B	109.5
C41—C40—H40B	109.1	C81—C82—H82C	109.5
C39—C40—H40B	109.1	H82A—C82—H82C	109.5
H40A—C40—H40B	107.8	H82B—C82—H82C	109.5
C42—C41—C40	112.0 (2)	C7—N1—C18	108.33 (16)
C42—C41—H41A	109.2	C7—N1—C6	125.95 (17)
C40—C41—H41A	109.2	C18—N1—C6	125.55 (16)
C42—C41—H41B	109.2	C24—N2—C32	108.32 (15)
C40—C41—H41B	109.2	C24—N2—C33	125.73 (16)
H41A—C41—H41B	107.9	C32—N2—C33	125.65 (16)
C41—C42—C43	114.7 (2)	C70—N3—C62	108.46 (15)
C41—C42—H42A	108.6	C70—N3—C71	125.77 (17)
C43—C42—H42A	108.6	C62—N3—C71	125.40 (18)
C41—C42—H42B	108.6	C45—N4—C52	108.65 (16)
C43—C42—H42B	108.6	C45—N4—C44	125.79 (16)
H42A—C42—H42B	107.6	C52—N4—C44	125.48 (16)
			
C1—C2—C3—C4	−173.8 (4)	C50—C51—C52—N4	−1.7 (2)
C2—C3—C4—C5	177.1 (3)	N4—C52—C53—C54	178.59 (17)
C3—C4—C5—C6	−174.6 (2)	C51—C52—C53—C54	−1.3 (3)
C4—C5—C6—N1	−175.59 (18)	C52—C53—C54—C55	5.9 (2)
N1—C7—C8—C9	177.7 (2)	C52—C53—C54—C60	−178.68 (16)
C12—C7—C8—C9	−0.2 (3)	C53—C54—C55—C56	−6.4 (3)
C7—C8—C9—C10	0.7 (3)	C60—C54—C55—C56	178.10 (16)
C8—C9—C10—C11	−0.4 (4)	C53—C54—C55—C57	170.33 (16)
C9—C10—C11—C12	−0.3 (3)	C60—C54—C55—C57	−5.2 (2)
C10—C11—C12—C7	0.8 (3)	C52—C51—C56—C55	2.7 (3)
C10—C11—C12—C13	−176.1 (2)	C50—C51—C56—C55	−179.17 (19)
N1—C7—C12—C11	−178.76 (18)	C57—C55—C56—C51	−174.69 (17)
C8—C7—C12—C11	−0.5 (3)	C54—C55—C56—C51	2.0 (3)
N1—C7—C12—C13	−1.1 (2)	C56—C55—C57—C58	172.32 (18)
C8—C7—C12—C13	177.16 (18)	C54—C55—C57—C58	−4.4 (3)
C11—C12—C13—C14	−6.1 (4)	C55—C57—C58—C59	5.7 (3)
C7—C12—C13—C14	176.7 (2)	C57—C58—C59—C64	−177.71 (18)
C11—C12—C13—C18	176.7 (2)	C57—C58—C59—C60	2.8 (3)
C7—C12—C13—C18	−0.5 (2)	C64—C59—C60—C61	−9.3 (3)
C18—C13—C14—C15	−2.0 (3)	C58—C59—C60—C61	170.19 (16)
C12—C13—C14—C15	−179.0 (2)	C64—C59—C60—C54	168.35 (16)
C13—C14—C15—C19	171.33 (19)	C58—C59—C60—C54	−12.1 (2)
C13—C14—C15—C16	−3.8 (3)	C53—C54—C60—C59	−162.14 (16)
C14—C15—C16—C17	7.9 (3)	C55—C54—C60—C59	13.2 (2)
C19—C15—C16—C17	−167.29 (18)	C53—C54—C60—C61	15.3 (3)
C14—C15—C16—C22	−176.38 (17)	C55—C54—C60—C61	−169.28 (16)
C19—C15—C16—C22	8.4 (3)	C59—C60—C61—C62	12.8 (2)
C15—C16—C17—C18	−6.2 (3)	C54—C60—C61—C62	−164.67 (16)
C22—C16—C17—C18	178.25 (17)	C59—C60—C61—C65	−162.12 (19)
C16—C17—C18—N1	179.89 (18)	C54—C60—C61—C65	20.4 (3)
C16—C17—C18—C13	0.4 (3)	C60—C61—C62—N3	176.86 (15)
C14—C13—C18—C17	3.7 (3)	C65—C61—C62—N3	−6.8 (2)
C12—C13—C18—C17	−178.46 (17)	C60—C61—C62—C63	−7.7 (3)
C14—C13—C18—N1	−175.79 (17)	C65—C61—C62—C63	168.67 (17)
C12—C13—C18—N1	2.0 (2)	N3—C62—C63—C64	172.90 (18)
C14—C15—C19—C20	−172.9 (2)	C61—C62—C63—C64	−1.8 (3)
C16—C15—C19—C20	2.2 (3)	C62—C63—C64—C59	5.7 (3)
C15—C19—C20—C21	−7.1 (3)	C60—C59—C64—C63	−0.1 (3)
C19—C20—C21—C26	−178.9 (2)	C58—C59—C64—C63	−179.57 (18)
C19—C20—C21—C22	1.0 (3)	C62—C61—C65—C66	−164.8 (2)
C26—C21—C22—C23	7.6 (3)	C60—C61—C65—C66	10.6 (4)
C20—C21—C22—C23	−172.25 (18)	C62—C61—C65—C70	5.02 (19)
C26—C21—C22—C16	−170.57 (18)	C60—C61—C65—C70	−179.59 (19)
C20—C21—C22—C16	9.6 (3)	C70—C65—C66—C67	3.3 (3)
C17—C16—C22—C21	161.48 (17)	C61—C65—C66—C67	172.3 (2)
C15—C16—C22—C21	−14.1 (3)	C65—C66—C67—C68	0.7 (3)
C17—C16—C22—C23	−16.5 (3)	C66—C67—C68—C69	−3.1 (4)
C15—C16—C22—C23	167.88 (17)	C67—C68—C69—C70	1.2 (3)
C21—C22—C23—C24	−12.6 (3)	C68—C69—C70—N3	−171.7 (2)
C16—C22—C23—C24	165.41 (17)	C68—C69—C70—C65	3.1 (3)
C21—C22—C23—C27	162.9 (2)	C66—C65—C70—N3	170.33 (16)
C16—C22—C23—C27	−19.0 (3)	C61—C65—C70—N3	−1.6 (2)
C22—C23—C24—N2	−175.91 (16)	C66—C65—C70—C69	−5.3 (3)
C27—C23—C24—N2	7.3 (2)	C61—C65—C70—C69	−177.26 (17)
C22—C23—C24—C25	9.5 (3)	N3—C71—C72—C73	−178.44 (18)
C27—C23—C24—C25	−167.33 (17)	C71—C72—C73—C74	179.86 (19)
N2—C24—C25—C26	−174.18 (18)	C72—C73—C74—C75	−178.1 (2)
C23—C24—C25—C26	−0.5 (3)	C73—C74—C75—C76	−176.5 (2)
C24—C25—C26—C21	−5.0 (3)	C77—C78—C79—C80	−179.0 (13)
C22—C21—C26—C25	1.3 (3)	C78—C79—C80—C81	168.2 (14)
C20—C21—C26—C25	−178.8 (2)	C79—C80—C81—C82	−146.1 (14)
C24—C23—C27—C28	164.8 (2)	C8—C7—N1—C18	−175.7 (2)
C22—C23—C27—C28	−11.2 (4)	C12—C7—N1—C18	2.4 (2)
C24—C23—C27—C32	−5.55 (19)	C8—C7—N1—C6	−0.2 (3)
C22—C23—C27—C32	178.5 (2)	C12—C7—N1—C6	177.95 (17)
C32—C27—C28—C29	−4.0 (3)	C17—C18—N1—C7	177.78 (18)
C23—C27—C28—C29	−173.6 (2)	C13—C18—N1—C7	−2.7 (2)
C27—C28—C29—C30	−0.3 (3)	C17—C18—N1—C6	2.2 (3)
C28—C29—C30—C31	2.9 (4)	C13—C18—N1—C6	−178.31 (17)
C29—C30—C31—C32	−0.9 (3)	C5—C6—N1—C7	−80.4 (2)
C30—C31—C32—N2	172.1 (2)	C5—C6—N1—C18	94.4 (2)
C30—C31—C32—C27	−3.7 (3)	C25—C24—N2—C32	168.07 (18)
C28—C27—C32—N2	−170.38 (16)	C23—C24—N2—C32	−6.2 (2)
C23—C27—C32—N2	2.0 (2)	C25—C24—N2—C33	−5.9 (3)
C28—C27—C32—C31	6.1 (3)	C23—C24—N2—C33	179.79 (16)
C23—C27—C32—C31	178.49 (17)	C31—C32—N2—C24	−173.74 (19)
N2—C33—C34—C35	176.94 (16)	C27—C32—N2—C24	2.5 (2)
C33—C34—C35—C36	−179.58 (16)	C31—C32—N2—C33	0.2 (3)
C34—C35—C36—C37	−178.04 (16)	C27—C32—N2—C33	176.47 (16)
C35—C36—C37—C38	177.19 (18)	C34—C33—N2—C24	81.7 (2)
C39—C40—C41—C42	65.1 (3)	C34—C33—N2—C32	−91.3 (2)
C40—C41—C42—C43	166.7 (2)	C69—C70—N3—C62	172.73 (19)
C41—C42—C43—C44	−179.0 (2)	C65—C70—N3—C62	−2.6 (2)
C42—C43—C44—N4	169.87 (17)	C69—C70—N3—C71	−0.6 (3)
N4—C45—C46—C47	−177.5 (2)	C65—C70—N3—C71	−175.95 (16)
C50—C45—C46—C47	−0.6 (3)	C63—C62—N3—C70	−169.25 (18)
C45—C46—C47—C48	−0.6 (3)	C61—C62—N3—C70	6.0 (2)
C46—C47—C48—C49	0.4 (4)	C63—C62—N3—C71	4.1 (3)
C47—C48—C49—C50	1.0 (3)	C61—C62—N3—C71	179.36 (16)
C48—C49—C50—C45	−2.1 (3)	C72—C71—N3—C70	92.2 (2)
C48—C49—C50—C51	174.9 (2)	C72—C71—N3—C62	−80.1 (2)
N4—C45—C50—C49	179.41 (18)	C46—C45—N4—C52	174.5 (2)
C46—C45—C50—C49	2.0 (3)	C50—C45—N4—C52	−2.7 (2)
N4—C45—C50—C51	1.6 (2)	C46—C45—N4—C44	−2.5 (3)
C46—C45—C50—C51	−175.81 (18)	C50—C45—N4—C44	−179.73 (17)
C49—C50—C51—C56	4.4 (4)	C53—C52—N4—C45	−177.17 (18)
C45—C50—C51—C56	−178.3 (2)	C51—C52—N4—C45	2.7 (2)
C49—C50—C51—C52	−177.3 (2)	C53—C52—N4—C44	−0.2 (3)
C45—C50—C51—C52	0.0 (2)	C51—C52—N4—C44	179.72 (17)
C56—C51—C52—C53	−3.2 (3)	C43—C44—N4—C45	82.9 (2)
C50—C51—C52—C53	178.23 (17)	C43—C44—N4—C52	−93.6 (2)
C56—C51—C52—N4	176.96 (16)		
